# Characterization of the complete chloroplast genome of Chinese privet *Ligustrum lucidum* (Oleaceae)

**DOI:** 10.1080/23802359.2018.1501298

**Published:** 2018-08-17

**Authors:** Rui-Hong Wang, Jing Gao, Meng-Di Li, Xue Wu, Chao Shen, Jun-Jie Wu, Ya-Qian Li, Yong-Hui Jin, Zhe-Chen Qi, Zong-Suo Liang

**Affiliations:** a College of Life Sciences, Zhejiang Sci-Tech University, Hangzhou, China;; b Zhejiang Province Key Laboratory of Plant Secondary Metabolism and Regulation, Hangzhou, China

**Keywords:** Oleaceae, Ligustrum, chloroplast genome, phylogenomics

## Abstract

*Ligustrum lucidum* is a species of privet native to the southern half of China. It is often used as an ornamental tree, sometimes as a cultivar. In present study, we reported the *Ligustrum lucidum* chloroplast (cp) genome. The total chloroplast genome size of *L. lucidum* was 154,793 bp. In total, 124 genes were identified, including 81 protein-coding genes, 8 rRNA genes, and 35 tRNA genes. Twenty genes contained introns (*clpP* and *ycf3* contained two introns) and 17 genes had two copies. The overall GC content of this genome was 38.2%. A further phylogenomic analysis of Oleaceae, including 21 taxa, was conducted for the placement of genus *Ligustrum*. The complete plastome of *L. lucidum* will provide a valuable resource for further genetic conservation, phylogenomic, and evolution studies of the genus and the family.

*Ligustrum* Linn. is a genus native to Asia, Australia, and Europe belonging the family Oleaceae, containing about 50 species (Qin 2009). Many members of the genus are grown as ornamental plants in parts of the world, known as privet. Several species of Chinese privet have become invasive species of nuisance in United States, New Zealand, and many other regions outside its range (Harrington and Miller 2005). *Ligustrum lucidum* is often used as an ornamental tree and has also become an invasive species in some areas where it has been introduced, such as urban areas in the southeastern United States. The seeds of *L. lucidum* are known as ‘*nu zhen zi’* in traditional Chinese medicine and are commonly used to nourish liver and kidney, treat tinnitus, vertigo, premature graying of the hair, and soreness of the lower back and knees (He et al. 2001; Lin et al. 2007). Here, we assembled and characterized the first complete plastome of genus *Ligustrum*. It will provide potential genetic resources for further evolutionary studies of the genus and the other relatives in Oleaceae.

Total DNA was extracted from fresh leaves of *Ligustrum lucidum* individual using DNA Plantzol Reagent (Invitrogen, Carlsbad, USA). It is collected from Baiyundong, Xuancheng, Anhui, China (30°37'39.63″N, 118°44'31.24″E, Voucher No. ZSTU01290, deposited at Zhejiang Sci-Tech University). The plastome sequences were generated using Illumina HiSeq 2500 platform (Illumina Inc., San Diego, CA, USA). In total, *ca*. 14.5 million high-quality clean reads (150 bp PE read length) were generated with adaptors trimmed. The CLC *de novo* assembler (CLC Bio, Aarhus, Denmark), BLAST, GeSeq (Tillich et al. 2017), and tRNAscan-SE v1.3.1 (Schattner et al. 2005) were used to align, assemble, and annotate the plastome.

The full length of *Ligustrum lucidum* chloroplast genome (GenBank Accession No. MH394207) was 154,793 bp and comprised of a large single-copy region (LSC with 86,418 bp), a small single-copy region (SSC with 17,957 bp), and two inverted repeat regions (IR with 25,209 bp). The overall GC content of the *L. lucidum* cp genome was 38.2% and the GC content in the LSC, SSC, and IR regions are 36.2, 32.9, and 43.6%, respectively. A total of 124 genes were contained in the cp genome (81 protein-coding genes, 8 rRNA genes, and 35 tRNA genes; 17 genes had two copies, which included 5 PCG genes (*ndhB*, *rpl2*, *rpl23*, *rps7*, and *ycf15*), 8 tRNA genes (*trnA-UGC*, *trnI-CAU*, *trnI-GAU*, *trnL-CAA*, *trnM-CAU*, *trnN-GUU*, *trnR-ACG*, and *trnV-GAC*), and all 4 rRNA species (*rrn4.5*, *rrn5*, *rrn16*, and *rrn23*). Among the protein-coding genes, 2 genes (*clpP* and *ycf3*) contained 2 introns, and other 10 genes (*atpF*, *ndhA*, *ndhB*, *petB*, *petD*, *rpl16*, *rpl2*, *rpoC1*, *rps12*, *rps16*) had 1 intron each.

Twenty-one chloroplast genome of Oleaceae were fully aligned with MAFFT v7.3 (Katoh and Standley 2013), and the maximum likelihood (ML) inference was performed using GTR + I + Γ model with 1000 bootstrap replicates with RAxMLv.8.2.1 (Stamatakis 2014) on the CIPRES cluster service (Miller et al. 2010). The result revealed that *L. lucidum* was most closely related to members of genus *Syringa* with the current sampling extent (Figure 1). The newly characterized *L. lucidum* complete chloroplast genome will provide essential data for further study on the phylogeny and evolution of the genus *Ligustrum* and the family Oleaceae.

**Figure 1.**
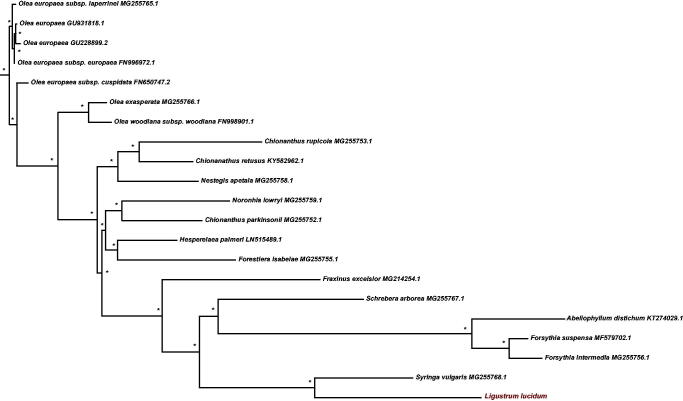
The best Maximum likelihood (ML) phylogram inferred from 21 chloroplast genomes in Oleaceae (bootstrap value are indicated on the branches, ‘*’ denotes a fully supported node).
